# Association between HIV status and Positive Prostate Biopsy in a Study of U.S. Veterans

**DOI:** 10.1100/tsw.2009.20

**Published:** 2009-02-15

**Authors:** Wayland Hsiao, Katrina Anastasia, John Hall, Michael Goodman, David Rimland, Chad W. M. Ritenour, Muta M. Issa

**Affiliations:** ^1^ Atlanta Veterans Affairs Medical Center, Emory University School of Medicine, Atlanta, GA, United States; ^2^ Department of Urology, Emory University School of Medicine, Atlanta, GA, United States; ^3^ Rollins School of Public Health, Emory University School of Medicine, Atlanta, GA, United States; ^4^ Division of Infectious Diseases, Emory University School of Medicine, Atlanta, GA, United States

**Keywords:** prostatic neoplasms, HIV, prostate needle biopsy, infectious diseases

## Abstract

HIV infection is associated with increased incidence of malignancies, such as lymphomas and testicular cancers. We reviewed the relationship between HIV infection and prostate cancer in a contemporary series of prostate biopsy patients. The study is a retrospective analysis of consecutive prostate biopsies performed at a VA Medical Center. The indications for performing a prostate biopsy included an abnormal digital rectal examination and/or an elevated PSA. Patients were categorized according to their HIV status, biopsy results, and various demographic and clinical characteristics. Univariate and multivariate analyses compared distributions of HIV status, and various clinical and demographic characteristics. The adjusted measures of association between HIV status and positive biopsy were expressed as odds ratios (ORs) and corresponding 95% confidence intervals (CI). The likelihood of positive biopsy was significantly higher among 18 HIV-positive patients compared to patients with negative HIV tests (adjusted OR = 3.9; 95% CI: 1.3–11.5). In analyses restricted to prostate cancer patients, HIV-positive patients were not different from the remaining group with respect to their prostate cancer stage, PSA level, PSA velocity, PSA density, or Gleason grade. There is an association between HIV infection and prostate biopsy positive for carcinoma in a population referred for urologic workup. Further confirmation of this association by prospective studies may impact the current screening practices in HIV patients.

## INTRODUCTION

It is estimated that over 33 million people worldwide and 1.2 million people in the U.S. are infected with human immunodeficiency virus (HIV). HIV infection has been convincingly shown to increase opportunistic infection, risk of prostatitis[1], and several malignancies, including testicular cancers, lymphomas, and penile cancers[2].

Advances in HIV treatment have resulted in greater longevity of HIV patients in the era of highly active antiretroviral therapy (HAART)[3]. With increasing age, it is expected that HIV-positive men will more likely reach older age when prostate cancer is more common in the general population. Although there have been previous reports of prostate cancer in HIV patients[4,5,6,7], it is currently unknown whether HIV infection predisposes to development of prostate cancer. Such information would have significant impact on prostate cancer screening and management in HIV patients. This retrospective study reviewed the relationship of HIV infection and prostate cancer in a large contemporary series of prostate biopsy patients.

## METHODS AND MATERIALS

The study is a retrospective analysis of a series of 2,052 consecutive veterans who had undergone transrectal ultrasound and prostate biopsy at the Atlanta VA Medical Center over a 5.5-year period (November 2000 to June 2006). Screening for prostate cancer is the standard of practice in the VA system for men aged 50 years or older. For those considered at higher risk for prostate cancer, such as African Americans and patients with positive family history, screening starts at an earlier age (40 years). The same criteria are used to screen male HIV patients who are followed in a primary care infectious disease clinic at the VA. The indications for prostate biopsy include abnormal digital rectal examination (DRE), elevated prostate serum antigen (PSA), or a combination of both.

Transrectal ultrasound (TRUS) of the prostate was performed under local anesthesia using the Logiq™ 200 Pro Series System (General Electric). The transverse (T), anteroposterior (AP), and longitudinal (L) dimensions of the prostate gland were measured to calculate the volume of the prostate using the standard ellipsoid formula as previously described[8,9].

Standard prostate biopsy was performed under ultrasound guidance using an 18-gauge needle with a Pro-Mag I 2.2 Md-tech spring-loaded biopsy device. The majority of the patients had eight to 12 biopsy cores; four to six cores from each prostate lobe. The biopsy cores were equally distributed from the apex, midregion, and base of the peripheral zone of each prostate lobe. When indicated particularly in substantially enlarged prostates, additional biopsy cores were obtained from the lateral aspect of the peripheral zone and from the transitional zone. A small and insignificant number of patients (<2%) underwent six-core biopsy earlier in the series.

The HIV status for each patient was categorized as positive, negative, or unknown (not tested). HIV-positive status was conferred if there was a positive ELISA followed by Western blot confirmation. HIV-negative patients had a negative ELISA test. Routine HIV screening is not utilized in our urology clinic and HIV testing was generally ordered by their primary care clinic. We did not inquire as to the reason for HIV testing.

Patient prostate biopsy results were either positive or negative for cancer. Patients whose only findings were high-grade prostatic intraepithelial neoplasia (HGPIN) were excluded from the analysis. Information on additional patient characteristics including age, race, family history, number of biopsy cores, prostate volume, and various PSA measures (prebiopsy level, density, and velocity) were obtained from medical records.

Univariate analyses used the Pearson chi-square test to compare biopsy-positive patients to biopsy-negative patients with respect to the distribution of HIV status, and various clinical and demographic characteristics. Multivariate logistic regression analyses evaluated the relationship between HIV status and positive biopsy, while adjusting for age, race, family history, and the number of biopsy cores. The results of the multivariate analyses were presented as adjusted odds ratio (OR), i.e., the ratio of the odds of a positive HIV test in a person with biopsy-confirmed prostate cancer to the odds of a positive HIV test in a similar person whose biopsy result was negative. All regression models were assessed for interaction, collinearity, and goodness of fit.

In a separate analysis limited to prostate cancer cases, HIV-positive and HIV-negative patients were compared with respect to their race and age, as well as with respect to various clinical characteristics including prostate cancer stage, grade, prostate volume PSA levels, PSA density, and PSA velocity. All analyses were performed using SPSS 13.0 for Windows (LEAD Technologies, Chicago, IL).

## RESULTS

Of the 2,052 prostate biopsies included in the dataset, 857 (42%) were positive for cancer and 1,065 (52%) were negative. The remaining 130 (6%) showed HGPIN and were excluded from analysis. Our final cohort of patients was comprised of 1,922 patients with a prostate biopsy either positive or negative for carcinoma (Table 1). We then analyzed the baseline characteristics of the patients stratified by the presence of prostate cancer. Patients with prostate cancer had a slightly higher median age (65 vs. 64, *p* = 0.01) as well as a higher PSA (7.8 vs. 5.4 ng/mL, *p* = 0.0003). Patients with prostate cancer were more likely to be African American, have a higher number of biopsies, and were more likely to be HIV positive. The percentage of patients with a family history of prostate cancer was similar in both groups.

**TABLE 1**
**Patients Profile Stratified by Prostate Biopsy Results**

| | | Total | Positive Biopsy | | Negative Biopsy | | *p* -value * |
|---|---|---|---|---|---|---|---|
| **Number of Patients** | | **1,922** | **857** | **100%** | **1,065** | **100%** | |
| **Race** | AA | 871 | 461 | 54.0% | 410 | 39.0% | **<0.01** |
| | Non-AA | 1,051 | 396 | 46.0% | 655 | 62.0% | |
| **HIV** | Positive | 18 | 14 | 1.6% | 4 | 0.4% | **0.02** |
| | Negative | 213 | 90 | 11.0% | 123 | 12.0% | |
| | Not Tested | 1,691 | 753 | 88.0% | 938 | 88.0% | |
| **Biopsy Cores** | Six (6) | 37 | 10 | 1.0% | 27 | 2.5% | **0.04** |
| | Eight (8) | 909 | 423 | 49.0% | 486 | 46.0% | |
| | Twelve (12) | 976 | 424 | 50.0% | 552 | 52.0% | |
| **Family History** | Positive | 359 | 173 | 20.0% | 186 | 18.0% | 0.13 |
| | Negative | 1,563 | 684 | 80.0% | 879 | 82.0% | |

* Based on Pearson chi-square test.

AA, African American.

A total of 18 patients in the overall series had HIV. All patients were on antiretroviral therapy at the time of prostate biopsy. Fourteen patients had biopsies positive for carcinoma and four patients had biopsies negative for carcinoma for a positive biopsy rate of 77.8% in HIV patients. In Table 2, duration of HIV infection, CD4 count, and viral load are shown for the 14 patients for whom the data were known. A total of 217 patients had a negative HIV test. Of the 217 HIV-negative patients, 94 patients had a biopsy positive for carcinoma for a positive biopsy rate of 43.3% in HIV-negative patients. The remainder of the patients did not have HIV results available and were considered “HIV unknown”.

**TABLE 2**
**HIV Profile of Biopsy Positive Patients**

| | Duration (years) | | CD4 | | Viral Load | |
|---|---|---|---|---|---|---|
| | *Median* | *Range* | *Median* | *Range* | *Median* | *Range* |
| **Positive Biopsy (n=11)** | **10** | 3 - 23 | **445** | 43 - 982 | **693** | <50 - 51,000 |
| **Negative Biopsy (n=3)** | **18** | 15 - 19 | **385** | 241 - 720 | **1,209** | 105 - 163,000 |

As shown in Table 3, the likelihood of positive biopsy was higher among HIV-positive patients when compared to HIV-negative patients (OR = 4.5; 95% CI: 1.5–14.4). This difference remained statistically significant (OR = 3.9; 95% CI: 1.3–11.5) after adjusting for age, race, family history, and number of biopsy cores. Similar results were observed when HIV-positive patients were compared to all other patients (those with a negative HIV test as “HIV unknown” patients) (adjusted OR = 3.5; 95% CI: 1.3–9.9).

**TABLE 3**
**Association between HIV Status and Prostate Biopsy Results**

| | | Positive Biopsy | Negative Biopsy | Odd Ratio | | Odd Ratio | |
|---|---|---|---|---|---|---|---|
| | | | | **Crude** | **95% CI** | **Adjusted** | **95% CI** |
| **HIV Tested** | HIV (+) | 14 | 4 | 4.6 | 1.5 - 14.4 | 3.9 | 1.3 - 11.5 |
| | HIV (-) | 94 | 123 | | | | |
| **All Patients** | HIV (+) | 14 | 4 | 4.4 | 1.4 - 13.4 | 3.5 | 1.3 - 9.9 |
| | All Other | 843 | 1,061 | | | | |

Analysis was then restricted to only those patients with biopsies positive for carcinoma (Table 4), comparing those patients with HIV vs. “all other” (HIV negative and HIV unknown) patients. HIV-positive patients were younger (mean age 56.7 vs. 65.0 years, $p = 0.01$), had smaller prostate gland volumes ($p = 0.04$), and included a significantly higher proportion of African Americans ($p = 0.02$). There was no significant difference between HIV patients and the overall group regarding prostate cancer clinical stage, PSA level, PSA velocity, PSA density, and Gleason grade. The rate of abnormal prostate on DRE (defined as asymmetry, induration, or nodule) was similar (53.3 vs. 52.7%, $p > 0.05$).

When the analysis was further restricted to the 14 HIV patients with biopsies positive for carcinoma compared to the 94 HIV-negative patients with biopsies positive for carcinoma, there was no statistical difference found in age, race, family history, prostate cancer clinical stage, grade, PSA, prostate volume, PSA density, and PSA velocity (data not shown).

**TABLE 4**
**Demographic and Clinical Analysis between HIV Status and Prostate Cancer Characteristics among Positive Biopsy Subjects (n = 857)**

| | | HIV (+) Patients | | All Other Patients | | *p* -value * |
|---|---|---|---|---|---|---|
| **Age** | 40 - 49 | 2 | 14.3% | 31 | 3.7% | **0.01** |
| | 50 - 59 | 8 | 57.1% | 239 | 28.4% | |
| | 60 - 69 | 2 | 14.3% | 299 | 35.5% | |
| | 70 - 79 | 2 | 14.3% | 216 | 25.6% | |
| | 80 - 89 | 0 | 0.0% | 58 | 6.9% | |
| **Race** | AA | 12 | 85.7% | 449 | 53.3% | **0.02** |
| | non-AA | 2 | 14.3% | 394 | 46.7% | |
| **Family History** | Positive | 5 | 35.7% | 168 | 19.9% | 0.10 |
| | Negative | 9 | 64.3% | 675 | 80.1% | |
| **Clinical Stage** | T0 - T1 | 7 | 50.0% | 397 | 47.1% | 0.83 |
| | T2 - T3 | 7 | 50.0% | 446 | 52.9% | |
| **Prostate Volume** (cc) | < 40 | 13 | 92.9% | 570 | 67.8% | **0.04** |
| | ≥ 40 | 1 | 7.1% | 271 | 32.2% | |
| **PSA** (ng/mL) | ≤ 4.0 | 0 | 0.0% | 91 | 10.8% | **0.38** |
| | 4.01 - 10.0 | 10 | 71.4% | 472 | 56.0% | |
| | > 10.0 | 4 | 28.6% | 280 | 33.2% | |
| **PSA Density** | < 0.15 | 1 | 7.1% | 221 | 26.3% | 0.09 |
| | ≥ 0.15 | 13 | 92.9% | 620 | 73.7% | |
| **PSA Velocity** | < 0.75 | 6 | 42.9% | 352 | 41.8% | 0.88 |
| | ≥ 0.75 | 8 | 57.1% | 491 | 58.2% | |
| **Gleason Grade** | ≤ 5 | 0 | 0.0% | 5 | 0.6% | 0.31 |
| | 6 - 7 | 14 | 100.0% | 729 | 86.5% | |
| | ≥ 8 | 0 | 0.0% | 109 | 12.9% | |

* Pearson chi-square test was used in the statistical analysis.

*Note:* HIV(+) patients, n = 14; all other patients, n = 843; except where data on two patients were not available for analysis (prostate volume and PSA density).

## DISCUSSION

In this study, we observed an association between HIV infection and prostate cancer risk. The study showed no significant difference in serum PSA and PSA density among HIV and non-HIV prostate cancer patients. This is consistent with earlier studies that showed that PSA levels are unrelated to HIV status[10], as well as studies by Pantanowitz et al. that showed that HIV severity does not seem to affect clinicopathological findings[11]. It is important to emphasize that the relationship between HIV infection and prostate cancer found in our study should be considered an association rather than a causative relationship.

Almost all patients in the HIV groups with biopsies positive for carcinoma were over 50 years old. There was only one "young" patient (44 years) who was both African American and had a positive family history for prostate cancer. As such, our data would not indicate a need to change biopsy guidelines in HIV patients. This is a critical question as the HIV population continues to age. The median age of HIV patients at our institution is 47 years, and the number of HIV patients over 50 years of age has tripled over the last 4 years at our institution.

It is of interest that most of our HIV patients with biopsies positive for carcinoma were African American. The rate of positive prostate biopsy for all African Americans in the overall series was 52.9% (461/871). This is in strong contrast to the 92.3% (12/13) positive prostate biopsy rate in African Americans who had HIV. Admittedly, this higher rate of positive biopsy in our HIV group may be due to a higher prevalence of African American patients. In the overall group, 45% (871/1922) of our population was African American, while 72% (13/18) of our HIV cohort was African American. Although small sample size does not allow any far-reaching conclusions, it does raise the question of whether HIV infection may even further raise the rate of prostate cancer in African Americans.

Our study has several important limitations. First, although we have found an association between HIV infection and prostate biopsy positive for carcinoma in our study, we have not shown an increased rate of prostate cancer in HIV-positive patients, which would require a large prospective cohort study. Second, the HIV status is unknown for most of our patients, although it is reasonable to assume that most of those who were not tested were, in fact, HIV negative. The exact HIV prevalence in our study group is unknown; however, one recent publication reported a 0.1–2.8% rate of HIV infection in the VA outpatient population[12]. Finally, it is certainly possible that our results were affected by referral bias. However, at our institution, we use the same criteria for urologic referral irrespective of HIV status.

Our study appears to be in line with previous observations indicating increased risk of other malignancies reported among HIV patients[13]. While both Patel et al. and Grulich et al. have published papers showing a lower incidence of prostate cancer in HIV patients[14,15], there is little information regarding age-specific incidence of prostate cancer in HIV patients. Prostate cancer prevalence for HIV patients may be lower when compared to the general populations because the HIV population is still a relatively young population and prostate cancer screening generally only begins at age 50. However, as the HIV population ages, there will be an increasing rate of prostate cancer in the HIV population. Age-specific incidence studies are needed. While limited by its retrospective nature and small numbers, our study is unique because it looks at an older HIV patient population with features concerning for prostate cancer. Sixteen of our 18 HIV-positive patients were over 50 years of age. Most significantly, we found that 14 of the 18 patients (78%) with indication for prostate biopsy had prostate cancer.

The mechanism underlying this association remains speculative, ranging from increased cytokine levels to decreased immune surveillance or direct effect of viral proteins. The role of infectious diseases in the etiology of prostate cancer remains debatable, particularly since we found no relationship between positive biopsy and either duration of HIV illness, CD4 count, or viral load.

The higher prevalence of HIV patients in the population, improved survival, and increased rate of positive biopsies for prostate cancer raises several issues for the urologic surgeon. Indications for potentially curable therapies must take into consideration the ultimate prognosis of the patient. Recent estimates indicate that the median survival after initiation of HAART is over 13 years[16]. Many cohort studies are reporting that most mortality in HIV patients is now due to non-AIDS causes. As such, the current literature supports aggressive therapy of early-stage prostate cancer in stable HIV patients with average life expectancy.

Although HIV-infected patients on antiretroviral regimens may provide complex management issues, these should not preclude considerations for radical surgical treatment when appropriately indicated. There is no compelling evidence that surgical complications are any worse in properly selected HIV patients who are stable on antiretroviral therapy[4,17,18].

## CONCLUSIONS

Our data may suggest that there is an association between HIV infection and prostate biopsy positive for carcinoma in a population referred for urologic workup. Further confirmation of this association by prospective studies may impact the current screening practices in HIV patients.
